# End-of-Season Influenza Vaccine Effectiveness Against Laboratory-Confirmed Influenza in Outpatient Settings, Beijing, China: A Test-Negative Design

**DOI:** 10.3390/vaccines13080809

**Published:** 2025-07-30

**Authors:** Jiaojiao Zhang, Zhaomin Feng, Ying Shen, Weixian Shi, Ying Sun, Jiachen Zhao, Dan Wu, Jia Li, Chunna Ma, Wei Duan, Jiaxin Ma, Yingying Wang, Lu Zhang, Xiaodi Hu, Quanyi Wang, Daitao Zhang, Peng Yang

**Affiliations:** 1 Beijing Key Laboratory of Surveillance, Early Warning and Pathogen Research on Emerging Infectious Diseases, Beijing Center for Disease Prevention and Control, Beijing 100013, China; elsa6066@163.com (J.Z.);; 2 Beijing Research Center for Respiratory Infectious Diseases, Beijing 100013, China; 3 School of Public Health, Capital Medical University, Beijing 100069, China

**Keywords:** influenza, vaccine effectiveness, influenza-like illness

## Abstract

This study aimed to estimate the end-of-season influenza vaccine effectiveness (VE) for the 2024/25 season in Beijing, China. **Methods:** We used a test-negative design (TND) to assess influenza VE among outpatients with influenza-like illness (ILI) enrolled through the influenza virological surveillance in sentinel hospitals in Beijing from week 44, 2024 to week 14, 2025. Cases were ILI patients who tested positive for influenza; controls were those who tested negative. **Results:** Among 18,405 ILI patients tested, 3690 (20.0%) were positive for influenza, with A(H1N1)pdm09 as the predominant strain (98.9%). The overall influenza vaccination coverage was 12.4%. Adjusted VE was 48.3% (95%CI: 40.4%–55.3%) against any influenza and 48.2% (95%CI: 40.3%–55.1%) against A(H1N1)pdm09, with the highest VE observed in adults aged 18–59 years (79.0%). The adjusted VE was similar for those vaccinated in 2023/24 only (53.1%) or both 2023/24 and 2024/25 seasons (50.8%), but lower for those vaccinated only in the 2024/25 season (48.5%). The adjusted VE was higher during the epidemic period (52.5%) than in the pre-epidemic (48.1%) and post-epidemic (35.3%) periods. **Conclusions:** Our findings indicate moderate VE against laboratory-confirmed influenza, especially A(H1N1)pdm09, during the end of the 2024/25 season in Beijing, China. Influenza vaccination provided protective effects across different epidemic periods. These timely estimates support ongoing public health communication and immunization strategies.

## 1. Introduction

Seasonal influenza vaccination remains a cornerstone of public health strategies to reduce the disease burden associated with annual influenza epidemics and to prepare for potential pandemics [1,2]. In the 2023–2024 season, influenza vaccination was estimated to have prevented 9.8 million illnesses, 4.8 million outpatient visits, 120,000 hospitalizations, and 7900 deaths in the United States alone [3]. To date, three types of influenza vaccine have been licensed: inactivated, live-attenuated, and recombinant hemagglutinin. Effectiveness analyses enable an evaluation of the beneficial impacts of influenza vaccines in real-world settings.

The influenza vaccine effectiveness (VE) is influenced by multiple factors, including the degree of virus strain matching, vaccination rates, and the immune background of individuals and groups, etc. Once vaccine strains are selected—typically based on global surveillance data—vaccine manufacturing requires after approximately six months. During this interval, genetic or antigenic changes in circulating viruses may lead to a mismatch and reduced VE [4]. To support timely public health decision-making and improve vaccine strain selection, early-season VE estimates have special value. Although early estimates are often based on small amounts of data and may be subject to bias, they provide crucial real-time information during the early stages of an epidemic. In January 2025, we reported early-season VE estimates for the 2024/25 influenza season in Beijing. Building on that analysis, we now present the first end-of-season assessment of influenza VE for the 2024/25 season.

This study is based on data from outpatient sentinel hospitals in Beijing to evaluate VE across different age groups, influenza virus types/subtypes, and epidemic phases. Notably, we divided the season into pre-epidemic, epidemic, and post-epidemic periods, allowing for comparison with earlier VE estimates and the assessment of VE estimation in different time windows. Our findings may contribute to the ongoing evaluation of influenza vaccine performance in real-world settings and offer timely evidence to inform strain selection and public health policy for upcoming seasons.

## 2. Materials and Methods

### 2.1. Subject Enrollment and Laboratory Detection

Influenza-like illness (ILI), defined as a temperature ≥38 °C with cough or sore throat, was enrolled in the Influenza Virological Surveillance System (IVSS) of Beijing between week 44, 2024 to week 14, 2025. The IVSS of Beijing was initiated in 2007 and currently includes 40 sentinel hospitals (Level II and above) and 19 network laboratories. According to the IVSS program, at least twenty pharyngeal swab specimens of medically attended influenza-like illness (ILI) cases were collected weekly by healthcare workers in outpatient settings and emergency departments in each sentinel hospital, and carried out throughout the year, namely about 1040 samples are taken in every sentinel hospital each year (52 × 20). To ensure the balance and randomness of ILI samples, 50% of the specimens must be completed in the first half of the week. Additionally, ILI cases ≥60 years should not be less than 10% of the total throughout the season. The inclusion criteria for subjects in the study were as follows: (1) sample collection occurring within seven days of onset; (2) the information regarding the onset, medical treatment, and testing of ILI patients is comprehensive; (3) a minimum interval of 14 days between vaccination and the onset.

Samples of ILI patients who visited within 3 days after the onset were priority collected by healthcare workers. The specimens were transported in a cool box at 4 °C for nucleic acid detection of influenza virus by reverse transcription quantitative polymerase chain reaction (RT-qPCR). Sequencing by synthesis of the viral hemagglutinin (HA) gene was undertaken on the PCR products of original specimens using the Illumina MiniSeq deep sequencing platform. A total of 91 influenza A(H1N1)pdm09 strains isolated between week 44, 2024 to week 14, 2025 were randomly selected and sequenced. Additional HA sequences were obtained from the Global Initiative for Sharing All Influenza Data (GISAID). Nucleotide sequences were assembled and aligned using MEGA software (version 5.0).

### 2.2. Study Design and Data Collection

The test-negative case–control study design was used to estimate VE against influenza of ILI patients in the outpatient and emergency department of IVSS. Cases were ILI patients who tested positive for influenza, and controls were influenza negative patients. The influenza epidemic threshold is 40% of the peak positivity of influenza in the current season, dividing the influenza epidemic into three periods: pre-epidemic, epidemic, and post-epidemic periods [5].

Epidemiological information of sampled ILI patients was collected by healthcare workers of the sentinel hospitals using a standardized electronic questionnaire, which included demographic characteristics (age, sex, region), the presence of chronic diseases, Influenza Status for the 2023/24 Season, clinical information (onset date, visiting date, symptoms, pneumonia), and the interval between the onset and the swab collection time. Chronic disease was defined as having any one of the following conditions: chronic obstructive pulmonary disease, asthma, cardiovascular disease, diabetes, immunodeficiency or organ transplant, renal impairment, rheumatologic disease, neuromuscular disease, cirrhosis or liver disease, neoplasms, autoimmune diseases, or hematological diseases. In the IVSS monitor plan, staff of collaborating laboratories were required to collect and integrate questionnaire data from sentinel hospitals and send datasets to the Beijing Center for Disease Prevention and Control (BJCDC).

The influenza vaccination status of sampled ILI patients was obtained from the Immunization Planning Information Management System. The influenza immunization plan commenced in mid-September 2024 across Beijing. According to the recommended immunization schedule of 2023–2024 in China [6], the influenza vaccines approved for marketing in China include trivalent or quadrivalent inactivated influenza vaccine (IIV3\IIV4), and trivalent live attenuated influenza vaccine (LAIV3). Among them, IIV has split vaccines and subunit vaccines, which can be used for vaccination in people ≥6 months, and are available in two dosage forms: 0.25 mL and 0.5 mL. LAIV3 is a freeze-dried preparation, intended for people aged 3 to 17, with each dose being 0.2 mL.

### 2.3. Statistical Analysis

Data were entered using the Etiological Surveillance System of Sentinel Hospitals in Beijing. Demographic and clinical characteristics of cases and vaccination were described using counts and percentages. Case and control statuses, as well as influenza vaccination status across groups, were compared by chi-square test. The unadjusted and adjusted VE were calculated using univariable and multivariable logistic regression models, respectively. These models compared the odds of influenza vaccination between influenza-positive cases and test-negative controls. Multivariable logistic regression models were adjusted for potential confounding factors including age, sex, region, epidemic period, chronic diseases, pneumonia, and interval between onset and sampling. The calculation formula of VE and 95% confidence interval (CI) is as follows: unadjusted and adjusted VE = (1 − unadjusted or adjusted odds ratios) × 100%, 95%CI for VE = (1 − CI_OR_) × 100%.

All data were analyzed using R version 4.3.3 software. All statistical tests were two-sided, with statistical significance established at a threshold of *p* < 0.05. The study was approved by the Institutional Review Board and Human Research Ethics Committee of Beijing Center for Disease Prevention and Control.

## 3. Results

### 3.1. Study Population and Influenza Activity in the 2024–2025 Season

Between 28 October 2024 (week 44) and 6 April 2025 (week 14), a total of 18,641 influenza-like illness (ILI) patients were enrolled through sentinel hospitals in Beijing. After excluding individuals with missing onset dates (n = 23), sampling delays beyond 7 days (n = 93), or recent influenza vaccination within 14 days prior to onset (n = 120), a total of 18,405 individuals were included in the TND analysis (Supplementary Figure S1). Among them, 3690 (20.0%) tested positive for influenza viruses (cases), while 14,715 (80.0%) tested negative (controls). The median age of the influenza-positive case group (38 years) was higher than the test-negative control group (28 years), and the median time interval from onset to sampling was 1 day (0–2 days) in both groups. Significant differences were observed between influenza-positive cases and test-negative controls in age group, region, chronic diseases, pneumonia, interval between onset and sampling, epidemic period, and consecutive season vaccination (all *p* < 0.05) (Table 1).

**Figure 1:**
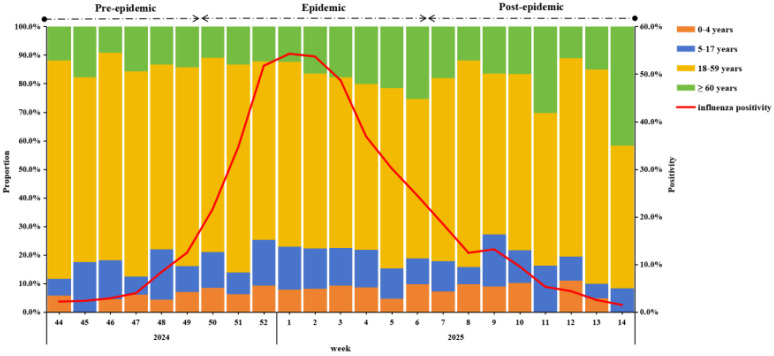
Weekly positivity and proportion among different age groups of influenza-positive specimens from IVSS in Beijing, China, 2024/25 season.

During the surveillance period, A(H1N1)pdm09 was the predominant strain (98.9%) (Supplementary Figure S1). Influenza activity began rising in week 44 of 2024, peaked in week 1 of 2025, and gradually declined thereafter. Using 40% of the current season epidemic peak as the threshold, the 2024–25 epidemic season was divided into three periods: a pre-epidemic period (weeks 44–49 in 2024), an epidemic period (weeks 50 in 2024 to week 6 in 2025), and a post-epidemic period (weeks 7–14 in 2025) (Figure 1).

In terms of the change in the positive proportion of age groups, the 18–59 group was always the highest, but from the second week of 2025, the proportion fluctuated downward, while the positive proportion of ≥60 years showed a fluctuating upward trend (Figure 1).

### 3.2. Phylogenetic Analysis

**Figure 2:**
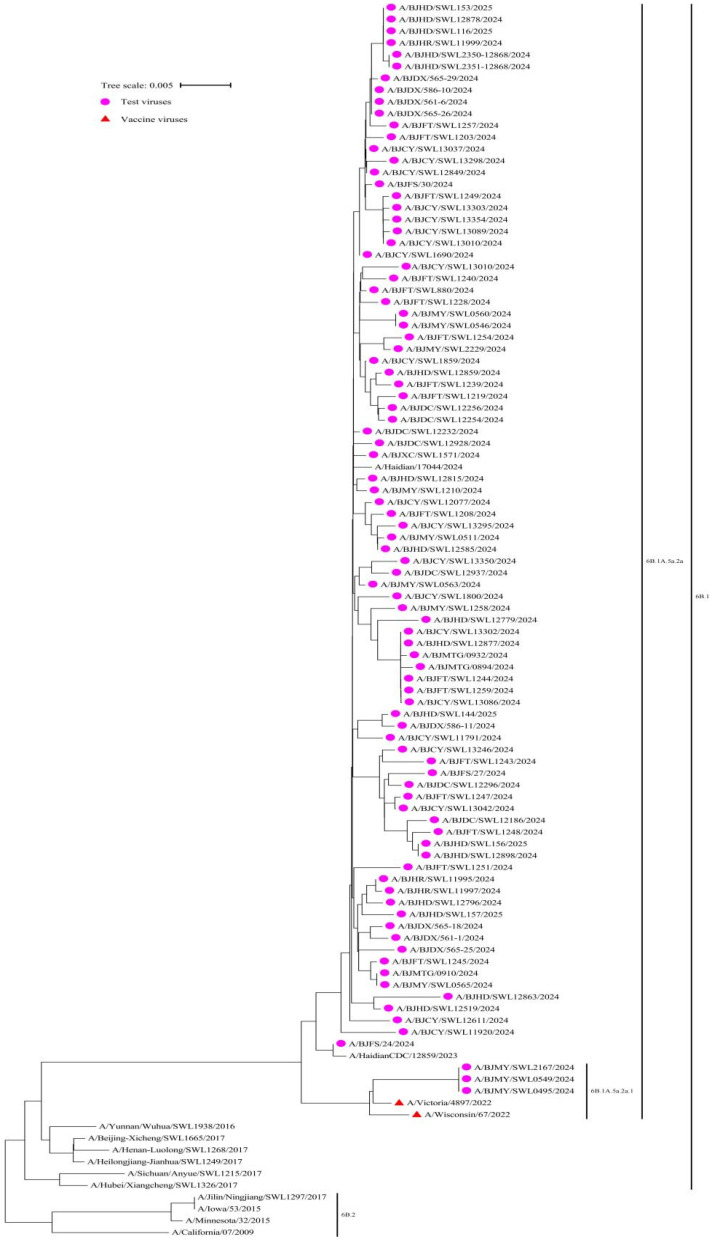
Phylogenetic analysis of the haemagglutinin (HA) gene of the influenza circulating A(H1N1)pdm09 virus strains (n = 91) compared to reference vaccine strains, Beijing, China, week 44 2024–week 14 2025.

We conducted phylogenetic analysis on influenza virus samples during the 2024–2025 season, in order to identify their genetic lineages. Among them, 100% (91/91) of the A(H1N1)pdm09 viruses belonged to the 6B.1A.5a.2a, with 3 viruses clustered in 6B.1A.5a.2a.1, and 88 in 6B.1A.5a.2a. All viruses exhibited a close genetic relationship with the reference vaccine strain A/Victoria/4897/2022 (H1N1) PDM09-like virus (clade 6B.1A.5a.2a.1) (Figure 2).

### 3.3. Influenza Vaccination Coverage and Vaccine Effectiveness Estimates

**Figure 3:**
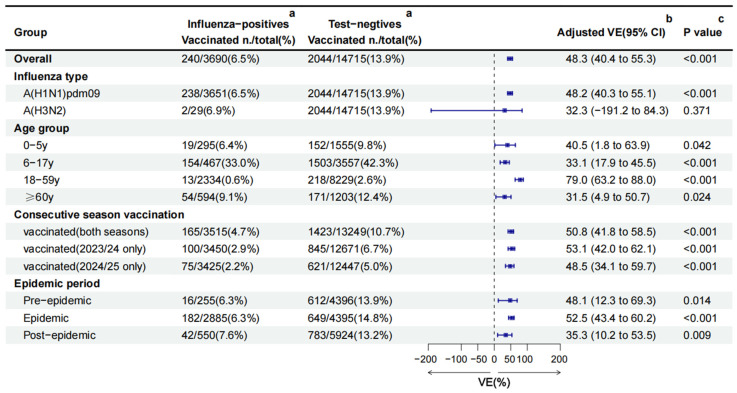
Influenza vaccine effectiveness estimated using the TND in the Beijing, China, 2024/25 season. CI: confidence interval; VE: vaccine effectiveness. **^a^** the number and rate of vaccination coverage in different groups. **^b^** The overall, A(H1N1)pdm09, A(H3N2), and consecutive season vaccination VE against influenza was obtained by adjusting for age group, sex, region, epidemic period, chronic diseases, pneumonia, and interval between onset and sampling. VE by age group was adjusted for sex, region, epidemic period, chronic diseases, pneumonia, and interval between onset and sampling. VE by epidemic period was adjusted for age group, sex, region, chronic diseases, pneumonia, and interval between onset and sampling. **^c^** a multivariable logistic regression model was used to compare the vaccination differences between the two groups on different factors.

Among the 18,405 ILI patients, 2284 (12.4%) had received influenza vaccination in the 2024/25 season. Nearly all vaccinations (99.7%, 2277/2284) were inactivated vaccines. Among 2277 inactivated vaccines, 86.4% (1967/2277) were IIV3, 13.6% (310/2277) were IIV4, and 97.8% (2226/2277) were split vaccines. Significant differences in vaccination coverage were observed in age group, sex, chronic diseases, and epidemic period (all *p* < 0.05) (Table 1). Vaccination coverage was highest in children aged 6–17 years (41.2%), followed by individuals ≥60 years (11.4%), 0–5 years (9.2%) and 18–59 years (2.2%). Vaccinated individuals constituted 6.5% (240/3690) of influenza-positive cases, compared with 13.9% (2044/14,715) among test-negative controls. This trend was consistent across age groups and epidemic periods (Figure 3).

The crude VE against all influenza was 56.9% (95%CI: 50.4% to 62.5%). After adjusting for age group, sex, region, epidemic period, chronic diseases, pneumonia, and the interval between onset and sampling, the overall adjusted VE was 48.3% (95%CI: 40.4 to 55.3) against all influenza. Subtype-specific adjusted VE was 48.2% (95%CI: 40.3 to 55.1) against A(H1N1)pdm09 and 32.3%(95%CI: −191.2 to 84.3) against A(H3N2) (Figure 3).

Stratified by age group, adjusted VE was highest in adults aged 18–59 years (79.0%, 95%CI: 63.2 to 88.0), followed by those aged 0–5 years (40.5%, 95%CI: 1.8 to 63.9), 6–17 years (33.1%, 95%CI: 17.9 to 45.5), and ≥60 years (31.5%, 95%CI: 4.9 to 50.7), respectively. Vaccination either in the current season or previous season offered comparable protection. The adjusted VE was 53.1% (95%CI: 42.0 to 62.1) among vaccinated only in 2023/24, 50.8% (95%CI: 41.8 to 58.5) among vaccinated in both seasons, and 48.5% (95%CI: 34.1 to 59.7) among vaccinated only in 2024/25 (Figure 3).

Between the different epidemic periods, the pre-epidemic (48.1%, 95%CI: 12.3 to 69.3) and epidemic (52.5%, 95%CI: 43.4 to 60.2) periods’ VE were similar, but higher than the post-epidemic period (35.3%, 95%CI: 10.2 to 53.5) (Figure 3).

## 4. Discussion

Since 2007, Beijing has launched a free influenza vaccination policy for the elderly (≥60 years) and school students (6–17 years) [7]. Compared with the 2023/24 season, influenza vaccine coverage in ILI patients (12.4% vs. 8.7%) and in children aged 6 to 17 years (41.2% vs. 25.9%) increased in the sentinel hospitals of Beijing during the end of 2024/25 season, but was significantly lower than other developed countries [8,9,10,11].

In this study, there are several points that require attention. Firstly, the influenza vaccine showed moderate effectiveness against all influenza (48.3%) and A(H1N1)pdm09 (48.2%) in Beijing during the end of 2024/25 season, which is similar to the early influenza VE of 2024/25 season in Beijing (48.5%), higher than the US Flu VE (42.0%) and lower than the Canadian (53.0%) for the early 2024/25 season [8,12,13]. The literature review by Rajaram, S states that influenza VE has a moderate protective effect of about 40% to 60% when vaccine strains are matched with circulating viruses, where characteristics of vaccinated persons and vaccine mismatch may be the two main factors affecting VE [14]. Vaccinator characteristics such as age are not controllable, but, in recent years, it has been identified that the adaptive growth and evolution of chicken embryo culture vaccines induce antigenic changes, increasing the risk of vaccine mismatch, and may further reduce the influenza VE, especially for the A(H3N2) influenza strain [15]. The early- and end-of-season influenza VE study in Beijing showed the close genetic relationship between the circulating influenza A(H1N1)pdm09 strains and the recommended vaccine strains [8]. In total, 421 (99.5%) A(H1N1)pdm09 viruses matched well with the vaccine, while 285 (58.0%) A(H3N2) viruses were well-matched from week 40 of 2024 to week 19 of 2025 in the United States [16]. Secondly, outpatients aged 18–59 years exhibited a higher VE compared to those aged 0–5 years, 6–17 years, and ≥60 years (79.0% vs. 40.5% vs. 33.1% vs. 31.5%). This was different from the early influenza VE of the 2024/25 season in Beijing (83.9% vs. 57.9% vs. 34.9% vs. 52.9%), which mainly manifested as follows: First, influenza VE decreased in all age groups, which may be related to the attenuation of antibodies with time [8,17]. Second, compared with the early influenza VE in the 2024/25 season in Beijing, the end-of-season influenza VE in the elderly (≥60 years) decreased significantly (52.9% vs. 31.5%), which might be related to the smaller number of positive cases in the elderly during the early 2024/25 season, leading to the unstable VE and a wide confidence interval [8]. Third, the heterogeneity in VE among different ages may be related to age-related differences in immune responses, such as the maturation of the immune system, the decline of immune function, and repeated vaccinations [18,19]. Fourth, in the case of low vaccination coverage in the adult (18–59 years, 2.2%), the highest VE in adults may be related to the high vaccination coverage in children (6–17 years, 41.2%). A Canadian study showed that herd immunity to influenza in children indirectly protected unvaccinated people in the community, reducing the overall incidence of influenza in the community by 61% [20]. Thirdly, the VEs for people vaccinated only in the 2023/24 season, vaccinated in both seasons, and vaccinated only in the 2024/25 season (53.8% vs. 50.8% vs. 48.5%) were similarly, which suggests that the vaccine composition from the previous season (A/Victoria/4897/2022 (H1N1)pdm09-like virus) may offer year-long protection due to its similarity with the circulating strains of the 2024/25 season [21,22,23,24].

The dynamic changes in influenza VE are the result of the combined effects of virus evolution, immune attenuation, and exposure risk [25,26]. In this study, the differences in influenza VE in the pre-epidemic, epidemic, and post-epidemic periods (48.1% vs. 52.5% vs. 35.3%) were statistically significant. According to the influenza vaccine production process and the changing characteristics of immune antibody levels, the influenza VE in different epidemic periods should have the following features [26,27]: Firstly, in the pre-epidemic, the antigenicity difference between the vaccine strain and the prevalent strain is relatively small, the antibody concentration of the vaccinators is relatively high, and the number of influenza infections is small, so the influenza VE should be higher. Secondly, in the epidemic period, the VE will fluctuate due to viral antigen variation, antibody level attenuation, differences in population immune status, and increased exposure intensity. However, the influenza VE in the epidemic period in this study had increased, which may be related to the vaccination policy of Beijing. Beijing launched an influenza immunization plan in mid-September 2024, in which children and the elderly were the priority groups [28]. Studies have shown that children play a role for both schools and the general public during influenza epidemics [6]. Therefore, it can be indirectly inferred that early childhood vaccination has a certain enhancing effect on the VE during the epidemic period. Thirdly, in the post-epidemic period, due to the continuous decline of antibodies and the weakening of immune memory, the influenza VE drops significantly.

The current analysis is subject to several limitations. First, participants were not selected randomly. The study recruited a similar number of ILI patients each week, and although ILI patients were required to be evenly distributed on a daily basis to minimize bias in the selection in the outpatient settings, there was centralized sampling on weeks with holidays. Second, as the predominant strain circulating during the 2024/25 season was A(H1N1) and none of the 20 ILI patients with B(Victoria) were vaccinated, it was not possible to estimate the VE against B(Victoria). Third, because the variables regarding age, sex, presence of chronic diseases, and date of symptom onset were collected by self-report questionnaire, the investigators were unable to verify the accuracy of responses, which may have led to reporting bias.

## 5. Conclusions

Our findings indicate moderate VE against laboratory-confirmed influenza, especially A(H1N1)pdm09, during the 2024/25 season in Beijing, China. Influenza vaccination provided protective effects across different epidemic periods. These timely estimates support ongoing public health communication and immunization strategies.

## Figures and Tables

**Table 1:** Characteristics of enrolled ILI patients and influenza vaccination status in Beijing, China, 2024/25 season.

Characteristic	Total, n (%)	Influenza-Positive Case, n (%)	Test-Negative Control, n (%)	*p* Value *^#^*	Vaccinated, n (%)	*p* Value *^#^*
**Overall**	18,405 (100.0%)	3690 (20.0%)	14,715 (80.0%)		2284 (12.4%)	
**Age group**						
0–5 years	1850 (10.1%)	295 (15.9%)	1555 (84.1%)	<0.001	171 (9.2%)	<0.001
6–17 years	4024 (21.9%)	467 (11.6%)	3557 (88.4%)		1657 (41.2%)	
18–59 years	10,563 (57.4%)	2334 (22.1%)	8229 (77.9%)		231 (2.2%)	
≥60 years	1968 (10.7%)	594 (30.2%)	1374 (69.8%)		225 (11.4%)	
**Sex**						
Male	8798 (47.8%)	1748 (19.9%)	7050 (80.1%)	0.558	1159 (13.2%)	0.003
Female	9607 (52.2%)	1942 (20.2%)	7665 (79.8%)		1125 (11.7%)	
Region *						
Urban	8190 (44.5%)	1717 (21.0%)	6473 (79.0%)	<0.001	1004 (12.3%)	0.463
Suburb	6530 (35.5%)	1186 (18.2%)	5344 (81.8%)		836 (12.8%)	
Exurban	3685 (20.0%)	787 (21.4%)	2898 (78.6%)		444 (12.0%)	
**Chronic diseases**						
Yes	1144 (6.2%)	273 (23.9%)	871 (76.1%)	0.001	74 (6.5%)	<0.001
No	17,261 (93.8%)	3417 (19.8%)	13,844 (80.2%)		2210 (12.8%)	
**Influenza Status for the 2023/24 Season**						
Uninfected	9457 (51.4%)	1951 (20.6%)	7506 (79.4%)	0.127	1197 (12.7%)	0.232
Infection	283 (1.5%)	54 (19.1%)	229 (80.9%)		27 (9.5%)	
Unclear	8665 (47.1%)	1685 (19.4%)	6980 (80.6%)		1060 (12.2%)	
**Pneumonia**						
Yes	912 (5.0%)	105 (11.5%)	807 (88.5%)	<0.001	104 (11.4%)	0.344
No	17,493 (95.0%)	3585 (20.5%)	13,908 (79.5%)		2180 (12.5%)	
**Interval between onset and sampling**						
≤3 days	17,531 (95.3%)	3552 (20.3%)	13,979 (79.7%)	<0.001	2175 (12.4%)	0.955
4–7 days	874 (4.7%)	138 (15.8%)	736 (84.2%)		109 (12.5%)	
**Epidemic period**						
Pre-epidemic	4651 (25.3%)	255 (5.5%)	4396 (94.5%)	<0.001	628 (13.5%)	0.002
Epidemic	7280 (39.6%)	2885 (39.6%)	4395 (60.4%)		831 (11.4%)	
Post-epidemic	6474 (35.2%)	550 (8.5%)	5924 (91.5%)		825 (12.7%)	
**Consecutive season vaccination**						
Vaccinated (both seasons)	1588 (8.6%)	165 (10.4%)	1423 (89.6%)	<0.001	1588 (100.0%)	-
Vaccinated (2023/24 only)	945 (5.1%)	100 (10.6%)	845 (89.4%)		0 (0.0%)	
Vaccinated (2024/25 only)	696 (3.8%)	75 (10.8%)	621 (89.2%)		696 (100.0%)	
Unvaccinated (both seasons)	15,176 (82.5%)	3350 (22.1%)	11,826 (77.9%)		0 (0.0%)	

Note: * Among the regions, urban contains six districts, namely Dongcheng, Xicheng, Haidian, Chaoyang, Fengtai, and Shijingshan; the suburban contains seven districts, namely Tongzhou, Shunyi, Changping, Daxing, Mentougou, Fangshan, and Jingkai; the exurban contains four districts, namely Pinggu, Miyun, Yanqing, and Huairou. **^#^** *p* values compare the influenza-positive case and negative control participants. The factor is calculated using chi-square test.

## Data Availability

The original database containing confidential patient information cannot be made publicly available. The anonymized data used in this study are available based on reasonable request to the corresponding author.

## Supplementary Materials

The following supporting information can be downloaded at: https://www.mdpi.com/article/10.3390/vaccines13080809/s1, Figure S1: Flow chart of subject enrollment in the TND for estimating influenza VE in Beijing, China, 2024/25 season.
