# Supplementary material for: End-of-Season Influenza Vaccine Effectiveness Against Laboratory-Confirmed Influenza in Outpatient Settings, Beijing, China: A Test-Negative Design
Source: Vaccines (Basel). 2025 Jul 30;13(8):809. doi: 10.3390/vaccines13080809 (PMC12390381; doi:10.3390/vaccines13080809)
Supplement: Supplementary file 1 [file vaccines-13-00809-s001.zip › vaccines-3723372-supplementary.pdf]

Supplementary

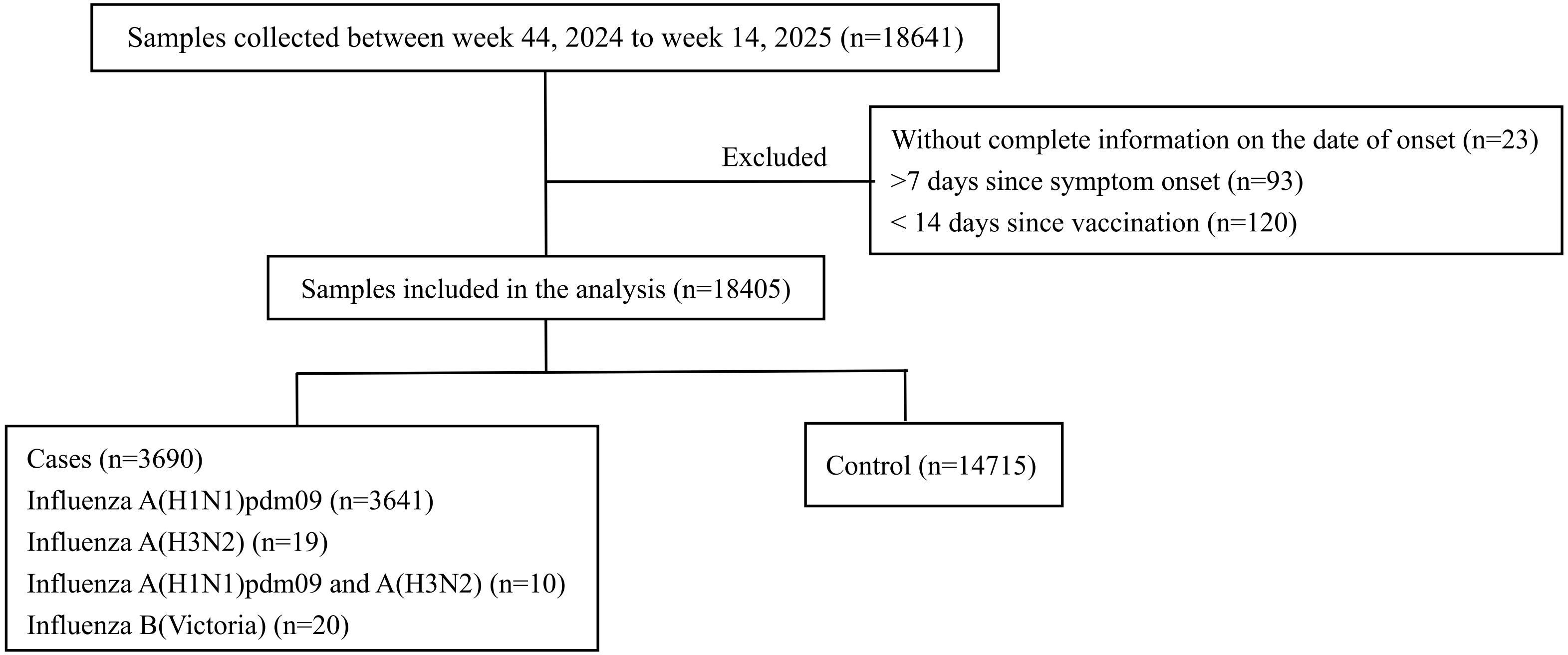

Fig.S1. Flow chart of subject enrollment in the TND for estimating influenza VE in Beijing, China, 2024/25 season.
